# Letter from Dr. Burt, Sec. T. S. M. A.

**Published:** 1886-04

**Authors:** W. J. Burt

**Affiliations:** Secretary T. S. M. A.


### SOCIETY NOTES.

EDITOR DANIEL'S TEXAS MEDICAL JOURNAL:

THE "Texas State Medical Association" will convene in Dallas, Texas, April 27th at 10 a. m.

There are many questions involved in the manner of conducting the proceedings, to make them pleasant as well as beneficial to each member. It is also well to be informed of important questions likely to come before the body for action. I propose briefly to allude to a few, only, of such matters in this letter.

1st. There is an amendment to the by-laws, pending, reducing the annual dues from $5 to $2.50. In my opinion it will be an unwise measure to adopt it, and would reduce our finances below a healthy standard.

Our association holds an honorable position among the sister societies, and it has largely been through our transactions that this place has been obtained. The articles themselves, and the beautiful and tasteful manner of presenting them, have received favorable comments from the profession all over the country. This could not have been accomplished with an empty treasury, and in my opinion the contemplated reduction of dues will cripple and retard our prosperity. I propose however to lighten the burden of obligations by abolishing one by-law and amending another.

1st. Strike out the by-law charging members of a local society one dollar annual dues. It is not right to charge a member an extra fee of $1 for the privilege of belonging to a local organization, because the tendency of this would prevent in a measure, the organizing of the profession into county societies and their affiliation with the State association.

2nd. Reduce the admission fees, to the State society to $2.50.

This will have a favorable influence in bringing more members into the association. To pay the present price of $5 admission fee and $5 annual dues prevents many good men from uniting with us.

3rd. The duties of the judicial council as now managed, are entirely too onerous. The members complain that they are deprived of nearly all the pleasure and benefit of the general sessions. I suggest the following plan as one way to partially remedy this.

The judicial council is composed of 12 members; my suggestion is, to divide the council into two committees. Take the first six on the list as committee No. 1, and the other six as committee No. 2.

Refer all applications for membership to committee No. 1. Refer all questions of an ethical and judicial character, and complaints and protests and credentials of delegates to committee No. 2. Each committee being smaller will do its work promptly, and any difficult or very important matter could be referred to a full council for adjudication.

4th. The judicial council requires applicants for membership to submit their diplomas with thier applications. This is a considerable hardship and annoyance, and I believe the same end could be accomplished by a modification of the rule so that a member of any local society, (whose constitution and by-laws require their members to be graduates and observe the code of ethics of the A. M. A., and which society is in affiliation with the State association;) could furnish a certificate, duly authenticated by the society, without requiring the old diploma to be produced.

5th. The committee on medical necrology, consisting of a member from each county, is and always has been, inefficient; and as a result of it, we have no biography of a number of deceased members. I propose that the association appoint an effiicient man to collect and present a brief biographical sketch of every deceased member, at our next annual meeting, and that the sum of $50 be donated for this work.

Hoping these suggestions will receive proper deliberation, I am yours truly,

W. J. Burt, M. D.
Secretary T. S. M. A.

